# Switching Assistance for Exoskeletons During Cyclic Motions

**DOI:** 10.3389/fnbot.2019.00041

**Published:** 2019-06-19

**Authors:** Nevio Luigi Tagliamonte, Simona Valentini, Angelo Sudano, Iacopo Portaccio, Chiara De Leonardis, Domenico Formica, Dino Accoto

**Affiliations:** ^1^Biomedical Robotics and Biomicrosystems Research Unit, Department of Engineering, Università Campus Bio-Medico di Roma, Rome, Italy; ^2^NEXT: Neurophysiology and Neuroengineering of Human-Technology Interaction Research Unit, Department of Medicine, Università Campus Bio-Medico di Roma, Rome, Italy; ^3^Robotics Research Centre, School of Mechanical and Aerospace Engineering, Nanyang Technological University, Singapore, Singapore

**Keywords:** assistive exoskeleton, adaptive controller, lower limb assistance, cyclic motions, series elastic actuator

## Abstract

This paper proposes a novel control algorithm for torque-controlled exoskeletons assisting cyclic movements. The control strategy is based on the injection of energy parcels into the human-robot system with a timing that minimizes perturbations, i.e., when the angular momentum is maximum. Electromyographic activity of main flexor-extensor knee muscles showed that the proposed controller mostly favors extensor muscles during extension, with a statistically significant reduction in muscular activity in the range of 10–20% in 60 out of 72 trials (i.e., 83%), while no effect related to swinging speed was recorded (speed variation was lower than 10% in 92% of the trials). In the remaining cases muscular activity increment, when statistically significant, was less than 10%. These results showed that the proposed algorithm reduced muscular effort during the most energetically demanding part of the movement (the extension of the knee against gravity) without perturbing the spatio-temporal characteristics of the task and making it particularly suitable for application in exoskeleton-assisted cyclic motions.

## 1. Introduction

Wearable exoskeletons are being developed for the therapy of patients undergoing neuro-motor recovery, for the daily-life support of subjects with permanent motor impairments and for the assistance of healthy individuals in industrial applications.

Human-robot mutual adaptation is a key mechanism to be considered in the control of wearable exoskeletons: robots have to synchronously adapt to the intended motion of the user, who in turn should be allowed to exploit robotic physical support to improve his motor function, in case of an impaired subject, or to possibly reduce the effort to perform a task, in case of a healthy subject.

These aspects strongly motivate the need to detect user's motion intention and to adjust robot action in a smooth, natural and non-constraining way.

Many approaches have been explored to assist human movements compliantly and adaptively, including interfaces for intention recognition based on biosignals (electroneurographic or electromyographic measurements) or on motion reconstruction/prediction (kinematic or dynamic measurements) (Tucker et al., 2015; Yan et al., 2015).

The first approach includes solutions that might be invasive, unreliable or sensitive to calibration, repeatability and signal acquisition/processing issues. This results in a complexity that can limit applicability to real scenarios. The second approach requires algorithms able to extract proper anticipatory information and to embed learning capabilities with a resulting high control effort, high computational load and need for additional sensors.

Interaction control schemes based on the measurement of limbs electromyographic (EMG) signals have been investigated for example in Kiguchi et al. (2003) and Lenzi et al. (2012). These controllers estimate the muscular force, and in turn the joint torques, from EMG measurements by using model-based (Cavallaro et al., 2006) or model-free (Kinnaird and Ferris, 2009) approaches, and assist the human with a fraction of the torques needed to perform the task. EMG-based solutions are sensitive to calibration procedure, electrodes positioning, skin condition and motion artifacts. Moreover, model-based torque estimation is computationally demanding, sensitive to subject anthropometry and to sensors placement. In some cases a good balance was found between complexity and performance, with a simplicity sufficient to operate in real-time applications (Cavallaro et al., 2006).

Another approach to predict limbs intended motion consists in extracting kinematic anticipatory information from different body districts by using wearable sensors. For example, the dynamical coupling between upper and lower body segments provides the appealing opportunity to extract anticipatory informative content on locomotion events from the arms oscillations (Novak et al., 2013). An alternative approach to intention recognition consists in directly observing joint kinematics. The strategy presented in Ronsse et al. (2011b) uses a non-linear model (based on Adaptive Frequency Oscillators, AFOs) able to intrinsically synchronize itself with human movements. An AFO is an oscillator whose amplitude, frequency and phase can adapt to an external input, such as human joint motions, thus reflecting real-time user's intention. This solution can be applied only in case of periodic motions.

Robotic physical assistance must be provided without forcing limbs toward harmful configurations and producing dangerous or painful forces. In this perspective traditional high-impedance position controllers may become inadequate and constraining, hence a proper modulation of human-robot interaction forces/torques is required. Based on intention recognition, indeed, assistance is usually produced by resorting to impedance controllers, i.e., by haptically delivering visco-elastic forces/torques to impart a reference motion trajectory, from which a deviation is comfortably allowed. Low values of stiffness and damping coefficients allow the user to deviate from the reference trajectory while high gains result in rigidly imparted movements. Despite a compliant delivery of assistive torques, impedance controllers provide physical support continuously and based on a pre-programmed equilibrium trajectory, with limited real-time control of the user over the machine. The effectiveness of combining AFO-based kinematic estimation and impedance control has been investigated in both upper and lower limb robots (Ronsse et al., 2011a; Tagliamonte et al., 2013).

Recently, a novel controller for gait assistance was developed in Wu et al. (2017), which calculates assistive hip and knee torques by using a reflex-based neuromuscular model, in particular by activating simulated muscle reflex loops based on gait state combining muscle-tendon dynamics. With this controller no pre-defined motor pattern is needed: ground contact detection is used to switch between stance and swing reflexes and joint angles are used to calculate simulated muscles state.

In this paper we propose a controller for assistive exoskeletons able to synchronize the action of the robot to the desired motion of a user during cyclic tasks by simply relying on basic kinematic information. The key idea behind the proposed *Switching Assistance Controller (SAC)* is to provide switching assistive inputs, i.e., to intermittently inject energy parcels into the human-robot system, to maintain a stable limit cycle, thus feeding the natural intrinsic oscillatory dynamics of the system with the minimum required amount of energy. This is quite crucial in applications where robots delivering assistive torques should avoid to potentially destroy natural efficient pendular motions.

Instead of rigidly imposing a predefined trajectory, the presented controller delivers intermittent assistive torques to produce functional motion and to minimize unwanted perturbations to the user's desired kinematic status. The SAC overcomes some of the limitations of the above mentioned approaches in that it does not provide continuous support, by only injecting energy parcels in short time windows, it does not need for complex mathematical models or high computational load, since it is based on a very simple control law, it does not require additional sensors, since it only uses kinematic information already present on the robotic joint, it does not need for biosignals, thus avoiding repeatability and usability issues, it automatically adapts in real-time to the user's intention without using predefined kinematic patterns, that might stereotype the motion, or learning approaches, that might introduce assistance delay due to adaptation time. While some of these advantages could be possibly achieved with model-based solutions, the complexity of human body action hinders these models from attaining the same benefits without complex algorithms.

An approach similar to SAC was pursued in Sugar et al. (2015), where a method to attain a limit cycle, by adding energy at resonance through a small and oscillatory torque signal, was developed based on a phase oscillator. Our control algorithm does not include phase oscillators but rather is based on the very simple idea of delivering an assistive torque in the phase when momentum is maximum and then induced velocity variations are minimum. What we present in this paper for robot-aided physical assistance is conceptually similar to what has been proposed in recent works to produce self-oscillations in a pendulum-based set-up (Aguilar et al., 2009), to excite and hold cyclic motions in a robotic arm embedding compliant actuated joints (Lakatos et al., 2014) or to induce resonance in a rotary parallel elastic actuator (Sudano et al., 2014). We validated experimentally a proof-of-concept of this novel controller by using a set-up including an exoskeleton to assist oscillations of the leg due to knee flexion-extension in the sitting position. Noticeably, similar simplified experimental conditions were used for controllers validation in other works such as Aguirre-Ollinger et al. (2007, 2012).

## 2. Switching Assistance Controller

Cyclic swinging motions of the legs are dynamic tasks whose efficient pendular nature may be destroyed by assistive robotic artifacts. Therefore, position, and even impedance or torque, controllers might alter tasks intrinsic dynamics thus resulting in unwanted increased muscle activation and metabolic cost for the user. Based on this observation we propose a controller, which is minimally invasive and capable of injecting into the human-robot compound dynamic system the minimum amount of energy needed to support oscillations and to enter a state of self-excitation. The basic idea is to control a robot without commanding pre-defined trajectories or torque profiles (even if adaptable to some user's intention detention rules), but rather inducing a functional motion, i.e., a motion which has some properties important to the functionality of a certain system, without the need of set-points tracking.

### 2.1. Basic Concept

A dissipative dynamic system can exhibit a limit cycle if a proper amount of energy is cyclically provided to feed oscillations. Energy can be conveniently injected in specific phases when the alteration to the system status can be minimized. As a simple example, we can imagine to administer energy into a 1-DOF damped pendulum with moment of inertia *J*. If the system has to be moved from an initial state identified by the angular velocity $\dot{q}\left(t\right)$ to a final state identified by the angular velocity $\dot{q}\left(t+\Delta t\right)$, in the moment when the kinetic energy is maximum (null potential energy), we have to provide the following amount of energy:

(1) $$\begin{array}{l} \Delta E=\frac{1}{2}J\left[\dot{q}{\left(t+\Delta t\right)}^{2}-\dot{q}{\left(t\right)}^{2}\right]≊J\dot{q}\left(t\right)\Delta \dot{q}\left(t\right) \end{array}$$

being the last approximation valid if $\dot{q}\left(t+\Delta t\right)≊\dot{q}\left(t\right)$.

Equation (1) indicates that, for a fixed amount of energy to be injected Δ*E*, the system status is minimally perturbed when angular momentum $J\dot{q}\left(t\right)$ is maximum since a minimum change of the initial velocity $\Delta \dot{q}\left(t\right)$ is achieved. Hence, the energy parcels can be conveniently injected in the time instant corresponding to maximum angular momentum to reduce perturbations to the system.

**Figure 1:**
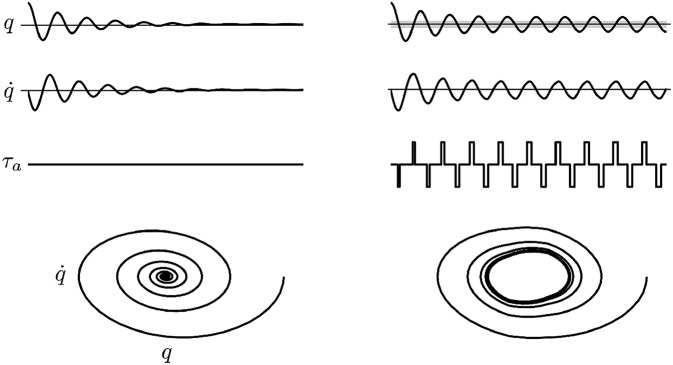
Example of the effect of the controller (2) on a damped pendulum. Angle *q*, velocity $\dot{q}$, torque applied by the controller τ_*a*_ and phase portrait are shown. Free oscillations of the damped pendulum (**Left**) and oscillations fed by the controller (**Right**). The gray area indicates the active region. The phase portrait shows that the controller is able to induce a limit cycle.

In the present paper we focused on simple 1-DOF oscillations of the leg around the knee joint axis of rotation (motion confined to the sagittal plane). Considering the leg as a pendulum that oscillates of an angle *q* around a stable equilibrium position *q*_0_, the proposed controller aims at providing an assistive torque τ_*a*_ concordant with the actual motion and in the neighborhood of *q*_0_, i.e., with a timing that minimally perturbs the system motion. Specifically, the assistive torque is delivered when the velocity during oscillation $\dot{q}$, and hence the angular momentum, is maximum. This can be mathematically expressed as:

(2) $$\tau_{a}=\{\begin{array}{ll} {\tilde{\tau }}_{a}\text{sgn}(\dot{q}) & \text{if }|q-q_{0}|\;\leq q_{w}\\ 0 & \text{else} \end{array}$$

where *q*_*w*_ is a symmetric angular window (hereafter called *active region*) where a constant assistive torque ${\tilde{\tau }}_{a}$ is provided. An example of the effect of the controller (2) is schematized in Figure 1, where the behavior of a damped pendulum is shown with and without the controller action.

### 2.2. Energy Modulation

To inject in the system enough energy to maintain the limit cycle, the amplitude of the assistive torque to be delivered within the active region can be selected as a function of the energy dissipated during the previous cycle.

**Figure 2:**
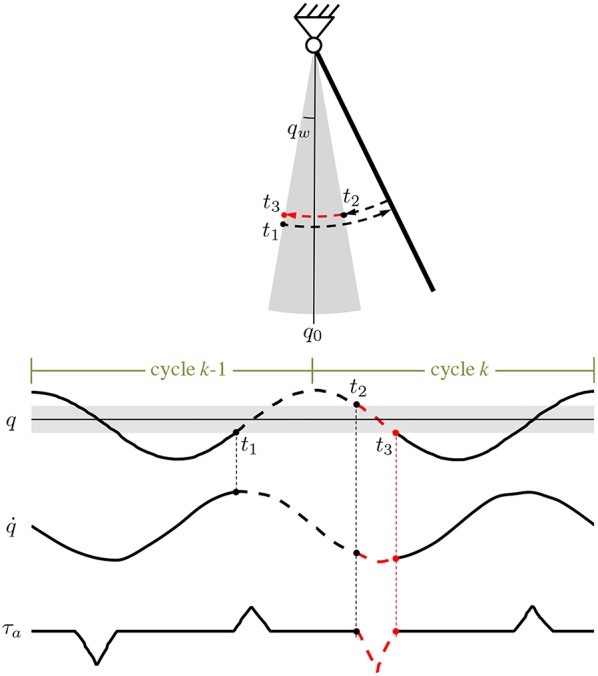
Schematic representation for the calculation of the dissipated energy and of the amplitude (3) for the assistance in the phase where $\text{sgn}\left(\dot{q}\right)<0$ (counterclockwise rotation is positive). In the time interval *t*_1_–*t*_2_ the lost energy is proportional to $\int_{t_{1}}^{t_{2}}\dot{q}dt$. The integral is calculated along the black dashed path and then released within the active region (gray area) during the time interval *t*_2_–*t*_3_ (dashed red path). The same calculation can be done for the amplitude (3) for the assistance in the phase where $\text{sgn}\left(\dot{q}\right)<0$.

For example, as schematized in Figure 2, for the assistance during the flexion phase ($\text{sgn}\left(\dot{q}\right)<0$), the energy dissipation is evaluated between *t*_1_, the time instant when the leg enters in the active region during the previous extension phase (*q* = −*q*_*w*_ and $\text{sgn}\left(\dot{q}\right)>0$) and *t*_2_, the time instant when the leg enters again in the active region during the flexion phase (*q* = *q*_*w*_ and $\text{sgn}\left(\dot{q}\right)<0$). The energy dissipated in the time interval *t*_1_−*t*_2_ is delivered within the active region during the time interval *t*_2_−*t*_3_, being *t*_3_ the time instant when the leg exits from the active region during the flexion phase (*q* = −*q*_*w*_ and $\text{sgn}\left(\dot{q}\right)<0$). Similarly, the representative time instants *t*_1_, *t*_2_ and *t*_3_ can be defined for the assistance in the extension phase. In formulas, the time instants are defined as:

- *t*_1_ | *q* = −*q*_*w*_ and $\text{sgn}\left(\dot{q}\right)>0$, *t*_2_ | *q* = *q*_*w*_ and $\text{sgn}\left(\dot{q}\right)<0$ and *t*_3_ | *q* = −*q*_*w*_ and $\text{sgn}\left(\dot{q}\right)<0$ for the assistance phase involving $\text{sgn}\left(\dot{q}\right)<0$
- *t*_1_ | *q* = *q*_*w*_ and $\text{sgn}\left(\dot{q}\right)<0$, *t*_2_ | *q* = −*q*_*w*_ and $\text{sgn}\left(\dot{q}\right)>0$ and *t*_3_ | *q* = *q*_*w*_ and $\text{sgn}\left(\dot{q}\right)>0$ for the assistance phase involving $\text{sgn}\left(\dot{q}\right)>0$.

considering counterclockwise rotation positive. Considering a viscous dissipation, assistive torque amplitude can be calculated as:

(3) $$\begin{array}{l} {\tilde{\tau }}_{a}^{t_{2}-t_{3}}=\frac{{\hat{\tau }}_{a}}{2q_{w}}\int_{t_{1}}^{t_{2}}{\dot{q}}^{2}dt \end{array}$$

In Figure 2 the path along which the dissipated energy is estimated is represented with a dashed black arrow, while the one showing the release of the assistive energy is represented with a dashed red arrow (the case of $\text{sgn}\left(\dot{q}\right)<0$ is reported). Of note, the amplitude (3) does not necessarily require the knowledge of the viscous friction coefficient, since it can just be considered as included in ${\hat{\tau }}_{a}$, which is a constant to be arbitrarily selected according to the desired assistance level.

### 2.3. Torque Amplitude Waveform

In implementing the SAC in the actual robotic device, described in section (3.1), we decided to slightly modify (2) to avoid discontinuities due the sign function, thus taking into account the limitations in the actuator closed-loop torque control bandwidth that did not allow to accurately track step commands with the frequency dictated by the SAC. Hence we substituted the constant ${\hat{\tau }}_{a}$ in (3) with a function of the angle *q* in the form:

(4) $$\begin{array}{l} {\hat{\tau }}_{a}\left(q\right)=2\tau_{a}^{*}\left(1-\frac{\left|q\right|}{q_{w}}\right) \end{array}$$

where $\tau_{a}^{*}$ is a constant representing the level of assistance.

Equation (4) allows to generate a desired triangular torque waveform, instead of rectangular one, still guaranteeing the same amount of delivered energy. The resultant pattern of the assistive torque is reported in Figure 2.

### 2.4. Assistance Timing

The idea behind the proposed control strategy is to deliver impulsive torques, i.e., to provide assistance in a time window short with respect to the cycle period. During preliminary tests we verified that each energy parcel was injected for a time that was on average around 15% of the duration of each motion cycle. The timing of torque delivery depended on the velocity of oscillation: fixed the amplitude of the active region (see Figure 2), the higher is the velocity of motion the shorter is the time in which the controller is active. Due to the short duration of the controller intervention, the specific torque waveform is expected not to have any significant impact on the performance of the controller.

## 3. Materials and Methods

### 3.1. Experimental Set-Up

**Figure 3:**
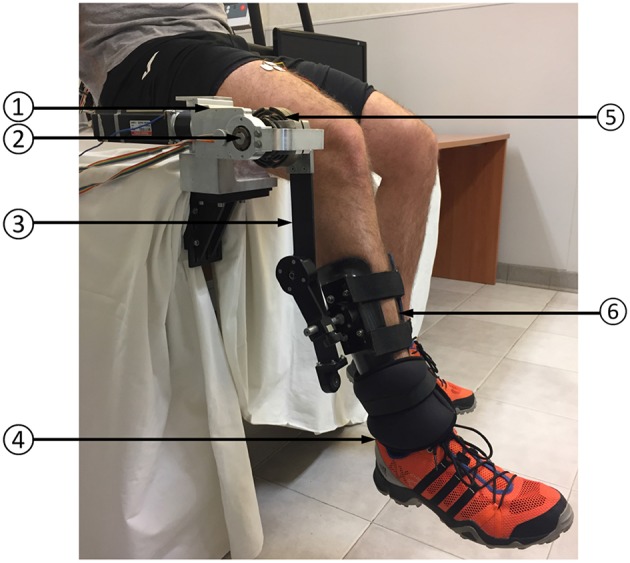
Experimental set-up: **(1)** SEA; **(2)** SEA axis of rotation; **(3)** link; **(4)** ankle load; **(5)** SEA spring (torque sensor); **(6)** leg cuff.

The assistive device used in this work is a 1-DOF knee exoskeleton actuated by a rotary Series Elastic Actuator (SEA) presented by the Authors in Accoto et al. (2013) and Accoto et al. (2014). The set-up is reported in Figure 3. The SEA includes a Maxon EC-4pole brushless DC motor (rated power: 300 W), a two-stage gearbox consisting of a planetary gear and a hypoid gear (reduction ratio: 64.5:1, efficiency: 76.5%) and a monolithic torsion spring (stiffness: 270.2 N m/rad) designed through an iterative FEM-based optimization process (Carpino et al., 2012). The spring deflection is measured by using two Gurley A10 absolute encoders (resolution: 1.9 · 10^−4^ rad) to calculate the interaction torque (resolution: 2.4 · 10^−2^ N m) as feedback signal for a closed-loop torque controller (see section 3.2). The SEA can deliver a continuous torque of 30 N m and a peak torque of about 60 N m. Its architecture allows placing the actuator stator alongside human thigh while aligning the output shaft to the human knee joint axis of rotation. An aluminum link, parallel to the human leg, connects the SEA shaft to a carbon fiber shank cuff. Anthropometric regulation is allowed by adjusting the relative position between the cuff and the link.

The control hardware consists of a Maxon EPOS2 70/10 unit running PID current control to drive the SEA motor (10 kHz) and a National Instruments compactRIO-9022 (cRIO), with a FPGA module and an embedded controller running LabVIEW Real Time (RT) software. The cRIO acquires SEA encoder signals (SSI communication, 10 kHz), transmits current set-points to the EPOS2 unit (CAN communication, 1 kHz) and runs the high-level controller (200 Hz). Two 4-channel amplifiers (QP522, Grass Technologies) are used to connect surface EMG electrodes (DENIS 5026, Spes Medica). EMG signals were band-pass filtered (10−1,000 Hz) and then acquired through a NI 9205 16-bit analog input module (sampling frequency: 2 kHz) integrated in the cRIO unit.

### 3.2. Control Implementation

**Figure 4:**
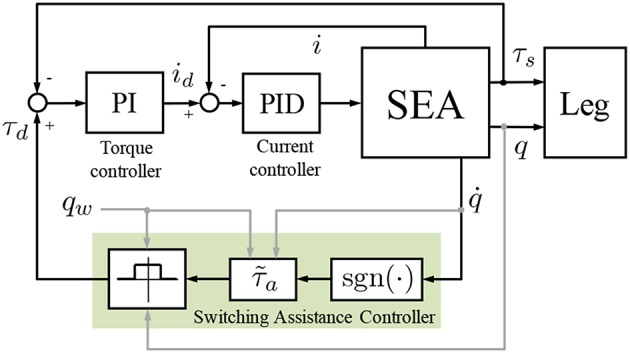
Block diagram of the controller. The SEA is torque-controlled based on a cascade approach (PID current controller nested in PI torque controller). *i*_*d*_ and *i* are the motor desired and actual currents, respectively. τ_*d*_ and τ_*s*_ are the SEA desired and actual torques, respectively. The SAC, indicated with the shaded area, is operational in the active region (*q*_*w*_ − *q*_0_) while the condition τ_*d*_ = 0 is guaranteed outside.

The proposed control scheme was implemented on the SEA based on a cascade approach similar to what proposed in Vallery et al. (2008) and Tagliamonte and Accoto (2013). In particular, a PI torque control loop, using as feedback the signal provided by the spring deflection measurement, was implemented on top of a low-level PID current control loop driving the motor. This approach allows regulating the interaction torque. Specifically, if a null desired torque τ_*d*_ is demanded to the actuator, transparency to the user's motion can be achieved. This mode is used here to minimize any interference to the natural oscillation dynamics of the human leg in the phases when assistance is not provided (i.e., outside the active region of the SAC). When assistance is provided by the SAC τ_*d*_ is commanded to be equal to τ_*a*_. This approach is represented in the block diagram of Figure 4. It is worth adding that the low-level PI torque controller is one of the mostly used in literature. This approach, despite very simple, is quite effective in our set-up since very low torques were delivered and basic single-pendulum dynamics were involved. It is expected that this solution may fail in more complex applications where higher demanded torques may create possible saturation effects, or where more complex dynamics may deteriorate the actuator transparent behavior due to parametric uncertainties and un-modeled dynamics. These issues, possibly raising in future more demanding applications, need to be tackled with more sophisticated control strategies like, for example, nonlinear schemes as proposed in Sun et al. (2018).

### 3.3. Experimental Protocol

The experimental protocol for the use of the robot was approved by the institutional Ethics Committee. Eight voluntary healthy subjects (4 men, 4 woman, right-handed, age: 25.25 ± 1.83, height: 1.73 ± 0.07 m, body mass: 72.63 ± 12.67 kg) were involved in the experiments. None of them had previously experienced the protocol adopted in this work. Each participant comfortably sat on a rigid surface and wore the robot on the dangling right leg. The stator of the SEA was framed to the sitting plane, the output shaft was aligned to the knee axis of rotation by adjusting the position of linear sliders. The robot cuff was fastened to each subject's leg. In the rest position the longitudinal axis of the leg was almost perpendicular to the floor. A 4 kg load was fastened to the ankle in order to increase the effort required to swing the leg, thus easing the detection of EMG signals and magnifying the benefit and effectiveness of the robotic assistance. During the tests, SEA torque and angle were measured. The activity of five major muscles responsible for knee flexion/extension motion were also recorded. The extensor muscles were: Rectus Femoris (RF), Vastus Lateralis (VL) and Vastus Medialis (VM); the flexor muscles were: Biceps Femoris (BF) and Semitendinosus (ST).

Each participant underwent two testing conditions, in the following order:

1. *Zero Torque* (ZT) mode. The robot was controlled with a null desired torque (τ_*d*_ = 0) not to perturb subject's motion and to set the baseline performance for each subject.
2. *Active Robot* (AR) mode. Assistance was provided only in the active region (τ_*d*_ = τ_*a*_); the robot was controlled in ZT mode outside the active region to minimize the perturbation of natural oscillatory dynamics.

The subjects were invited to oscillate their leg in the most comfortable and effortless way. The rotation angle was shown on a PC screen in front of the subject by means of a digital indicator needle. Subjects were asked to swing their leg so that the indicator needle could oscillate within an angular window shown on the same screen by means of two limiters.

Each experimental session included two phases:

1. *Familiarization*: 5 min in ZT mode and 5 in AR mode, to get used to the robot and identify the most comfortable swinging motions.
2. *Testing*: 3 subsequent tests, interrupted by 30 min of resting periods, consisting in 5 min of knee oscillations in ZT mode and 15 min in AR mode.

The angle limits on the screen were set to [-31, 19] deg (conventionally considering positive the flexion motion) and the active region was set to *q*_*w*_ = 10 deg. The level of assistance in equation (4) was experimentally selected considering a trade-off between comfort and perceived physical support. This value was set based on the outcomes collected in preliminary pilot tests performed on a reduced group of subjects in which different conditions/parameters were assessed in terms of EMG signals reduction. These tests were also useful to identify the proper duration of AR mode task as a trade-off between the time required to the subject to adapt to the imparted robotic torque field and the duration inducing undesired fatigue effects.

During preliminary pilot experiments we tested on a group of three subjects (1 man, 2 woman, right-handed, age: 25.33 ± 1.15, height: 1.67 ± 0.03 m, body mass: 67.00 ± 12.12 kg) three different assistance levels, i.e., setting $\tau_{a}^{*}$ to 1.0 N m, 1.5 N m and 2 N m (low, medium and high assistance). For each assistance level, subjects underwent a familiarization trial and then three different measurement trials, each composed of 4.5 min of task in ZT mode and of 4.5 min of task in AR mode. We could verify that the medium assistance level could decrease the EMG activity of most of the muscles while low and high assistance levels had a more heterogeneous effect and, in many cases, caused an increase of the muscular activity. Hence, in these two cases the robotic aid had forced somehow the subject to react in a way that increased his effort in performing the task. The value $\tau_{a}^{*}=1.5$ N m was then selected for further experiments reported within this paper. This value provided a physical support considered comfortable for the analyzed subjects while still showing, as a proof of concept of the presented approach, the capability of reducing the effort needed to perform the selected task in terms of EMG activity reduction.

In the final test, the experimental conditions (i.e., the assistance level) were blinded to the subjects, in order not to bias the results.

### 3.4. Data Processing

#### 3.4.1. Cycles Selection Based on EMG

Pre-filtered EMG signals were full-wave rectified and low-pass filtered by using a zero-lag second-order Butterworth filter to calculate the envelope. A cut-off frequency of 5 Hz was selected similarly to other works (e.g., Lenzi et al., 2013; Tagliamonte et al., 2014). Each oscillation cycle *k* was isolated by using a peak detection algorithm on the angle data and flexion-extension phases were identified based on the sign of the angular velocity. Knee angle *q*, angular velocity $\dot{q}$, assistive torque τ and EMG signals *M* were segmented based on flexion-extension cycles identification and were temporally normalized with respect to the maximum cycle duration. EMG artifacts, due to accidental cables movements or to pressures inadvertently exerted by the users on the electrodes, were removed using the iterative algorithm described below. First of all, acquired data streams were segmented by dividing the cycles in ZT condition from those in AR condition. Then, each of the two segments was further divided in a sequence of 2.5 min windows, sequentially ordered, in order to possibly highlight relevant trends in muscular activity. Two windows were obtained for the ZT condition (overall duration in ZT: 5 min) and 6 windows for the AR condition (overall duration in AR: 15 min).

The algorithm for artifacts removal consisted, for each of the five analyzed muscles and for each of the 8 windows, of the following iterative steps:

1. calculation of the RMS value ($M_{k}^{RMS}$) for each *k*-th cycle and calculation of the mean value (${\bar{M}}^{RMS}$) and SD value (${\bar{\bar{M}}}^{RMS}$) among all the cycles;
2. calculation of $d_{k}=|M_{k}^{RMS}-{\bar{M}}^{RMS}|$;
3. discarding data (muscular activity, joint angle, angular velocity and assistive torque) related to k^*^−th cycle for which $d_{k^{*}}$ value is maximum and greater than $3{\bar{\bar{M}}}^{RMS}$.
4. If data were discarded at step (3), restart from (1).

In 80% of the 24 performed tests (8 subjects, 3 sessions) the cycles excluded due to EMG artifacts were less then 10% of total. In the remaining cases, the percentage of removed cycles felt in the range 10–25% due to particularly noisy signals of flexion muscles caused by mechanical artifacts. For each subject, and in each condition, the total number of flexion/extension cycles for the final analysis was in the order of 1,000.

#### 3.4.2. Extraction of Relevant Features

To extract the features of interest we considered the following subsets of quantities: $\chi =\left\{M,q,\dot{q},\tau \right\}$ and $\psi =\left\{M,\dot{q}\right\}$. Testing modes will be identified with *m* = {*ZT, AR*} while the letter ϕ = {*fc, fl, ex*} will be used to indicate if a certain quantity is calculated over the full cycle (*fc*), during flexion phase (*fl*) or during extension phase (*ex*). *k* will indicate the generic flexion-extension cycle and *N*_*m*_ the total number of cycles in mode *m*.

The following relevant quantities were calculated for further analysis:

- Mean of χ among all the cycles, expressed as:
  (5) $$\begin{array}{l} {\bar{\chi }}_{m}=\frac{\sum_{k=1}^{N_{m}}\chi_{k,m}}{N_{m}} \end{array}$$
- Mean RMS value of the angular velocity in the mode *m* and in the phase ϕ, expressed as:
  (6) $$\begin{array}{l} {\bar{\dot{q}}}_{m-\phi }^{RMS}=\frac{\sum_{k=1}^{N_{m}}{\dot{q}}_{k,m-\phi }^{RMS}}{N_{m}} \end{array}$$
- Percentage variation of the mean RMS value of the angular velocity in AR mode with respect to ZT mode, in phase ϕ, expressed as:
  (7) $$\begin{array}{l} \Delta {\bar{\dot{q}}}_{\phi }^{RMS}=\frac{{\bar{\dot{q}}}_{ZT-\phi }^{RMS}-{\bar{\dot{q}}}_{AR-\phi }^{RMS}}{{\bar{\dot{q}}}_{AR-\phi }^{RMS}}100 \end{array}$$
- EMG activation in AR mode with respect to the ZT mode in phase ϕ, expressed as:
  (8) $$\begin{array}{l} \alpha_{\phi }=\frac{\sum_{k=1}^{N_{AR}}\left(M_{k,AR-\phi }^{RMS}\right)}{\sum_{k=1}^{N_{ZT}}\left(M_{k,ZT-\phi }^{RMS}\right)}\frac{N_{ZT}}{N_{AR}} \end{array}$$
- EMG activation in AR mode with respect to the ZT normalized with respect to the angular velocity, in phase ϕ, expressed as:
  (9) $$\begin{array}{l} \beta_{\phi }=\frac{\sum_{k=1}^{N_{AR}}\left(M_{k,AR-\phi }^{RMS}/{\dot{q}}_{k,AR-\phi }^{RMS}\right)}{\sum_{k=1}^{N_{ZT}}\left(M_{k,ZT-\phi }^{RMS}/{\dot{q}}_{k,ZT-\phi }^{RMS}\right)}\frac{N_{ZT}}{N_{AR}} \end{array}$$

#### 3.4.3. Torque Normalization

Assistive torque was normalized with respect to the maximum gravitational torque calculated as:

(10) $$\begin{array}{l} \tau_{g}=g\left(l_{com}m_{limb}+l_{leg}m_{load}\right) \end{array}$$

where *g* is gravity, *m*_*limb*_ = 0.061*m* is the mass of the limb (leg plus foot), *l*_*com*_ = 0.173 *h* is the distance of the limb center of mass from the knee joint along the leg axis, *l*_*leg*_ = 0.246*h* is the leg length (distance from the knee joint to the ankle joint) and *m*_*load*_ = 4 kg is the mass of the load fastened to the ankle. Anthropometric data for each subject of mass *m* and height *h* estimated based on Winter (2009). The assistive torque during flexion and extension felt in the range 6.5−18% of τ_*g*_ and 12.8−20% of τ_*g*_, respectively.

#### 3.4.4. Delivered Energy

To estimate the total energy associated with leg plus load oscillations, we calculated the peak value of the kinetic energy (i.e., the value corresponding to a null potential energy). For each *k*-th cycle the kinetic energy was $Ke_{k}=\frac{1}{2}J_{tot}{\dot{q_{k}}}^{2}$, in which *J*_*tot*_ = *J*_*leg*_ + *J*_*load*_ is the total moment of inertia calculated as the sum of moment of inertia of the leg and of the load. In particular, $J_{leg}=r_{leg}^{2}m_{leg}$ and $J_{load}=l_{leg}^{2}m_{load}$. From Winter (2009) we could retrieve that the leg radius of gyration with respect to the knee joint axis of rotation was *r*_*leg*_ = 0.735*l*_*leg*_. Moreover, for each single action of the controller, the energy delivered by the actuator was calculated as $Ke_{k}=\int_{t_{2}}^{t_{3}}\tau_{a}dq$. The controller energy contribution ϵ_*k*_ was finally calculated as the amount of energy delivered by the actuator with respect to the net energy involved in the movement, i.e., $\epsilon_{k}=\frac{De_{k}}{Ke_{k}^{max}}100$.

#### 3.4.5. Curves Cross-Correlation

Cross-correlation coefficients between the mean curves in ZT and AR mode were evaluated on the subset $\xi =\left\{M,q,\dot{q}\right\}$ as follows:

(11) $$\begin{array}{l} \rho \left({\bar{\xi }}_{AR},{\bar{\xi }}_{ZT}\right)=\frac{\text{cov}\left({\bar{\xi }}_{AR},{\bar{\xi }}_{ZT}\right)}{{\bar{\bar{\xi }}}_{AR}{\bar{\bar{\xi }}}_{ZT}} \end{array}$$

being cov(·, ·) the covariance and $\bar{\bar{\cdot }}$ the standard deviation. Cross-correlation coefficients were classified in *K* levels calculated through Sturges' formula:

(12) $$\begin{array}{l} K=\left\lceil \log_{2}T\right\rceil +1 \end{array}$$

In (12) *T* indicates the number of tests, i.e., the number of comparisons between the quantities ${\bar{\xi }}_{AR}$ and ${\bar{\xi }}_{ZT}$. 24 tests were performed to measure joint angle and velocity (3 sessions on 8 subjects); 120 tests were performed to measure EMG signals (5 muscles, 8 subjects, 3 sessions), of which 72 for the 3 extensor muscles and 48 for the 2 flexor muscles (i.e., 24 tests per muscle).

#### 3.4.6. Statistical Analysis

The effect of the proposed controller on the muscular activity was evaluated based on the quantities (α_ϕ_ and β_ϕ_) defined in (8) and (9). The controller is effective in reducing muscular activity if α_ϕ_ and β_ϕ_ are less than 1.

This condition is meaningful only if such quantities differ from 1 in a statistically significant manner. Given the distributions $\zeta_{m-\phi }=\left\{M_{k,m-\phi }^{RMS},M_{k,m-\phi }^{RMS}/{\dot{q}}_{k,m-\phi }^{RMS}\right\}$ (with *k* = 1, …, *N*_*m*_) the chi-squared normality test was performed as a test decision for the null hypothesis that the data analyzed ζ_*k*_ came from a normal distribution with a mean and variance estimated from that data distribution. After this test, the following cases were derived:

- For both ζ_*AR*−ϕ_ and ζ_*ZT*−ϕ_ the null hypothesis was not rejected. A *t*-test was performed that returned a test decision for the null hypothesis that the data in ζ_*m*−*t*_ came from independent random samples from normal distributions with equal mean values and equal but unknown variance values.
- For at least one between ζ_*AR*−ϕ_ and ζ_*ZT*−ϕ_ the null hypothesis was rejected. A non-parametric test, namely the Wilcoxon test, was performed, whose null hypothesis is that the probability of an observation from the distribution ζ_*AR*−ϕ_ exceeding an observation from the distribution ζ_*ZT*−ϕ_ equals the probability of an observation from ζ_*ZT*−ϕ_ exceeding an observation from ζ_*AR*−ϕ_.

If the null hypothesis was rejected, we considered admissible to use (8) and (9) to classify cases of increase or decrease of muscular activity in AR mode with respect to ZT mode, otherwise the variation was classified as Not Statistically Significant (NSS).

## 4. Results

**Figure 5:**
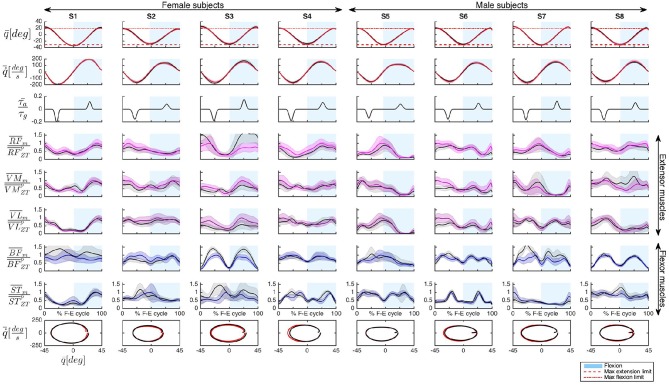
Representative data of the 8 subjects (S1–S8) recorded during session 3. The following quantities have been represented organized by rows: mean knee angle $\bar{q}$, mean knee angular velocity $\bar{\dot{q}}$, mean assistive torque normalized with respect to the maximum gravitational torque ${\bar{\tau }}_{a}/\tau_{g}$, and mean EMG activity, ${\bar{M}}_{m}$ (with *m* = {*AR, ZT*}) of extensor muscles (*RF*, *VM*, *VL*) and flexor muscles (*BF*, *ST*), normalized with respect to the peak of the muscle activity in ZT mode ${\bar{M}}_{ZT}^{p}$. Flexion is conventionally indicated as positive. Shaded regions indicate the standard deviation. In the last row the phase portrait in AR and ZT modes is represented. Time normalization is done with respect to the Flexion-Extension (F-E) cycle duration.

To validate the effectiveness of the controller, AR mode and ZT mode were compared in terms of knee joint angle *q*, angular velocity $\dot{q}$ and muscular activity of three extensor muscles (*RF*, *VM*, *VL*) and two flexor muscles (*BF*, *ST*). Figure 5 includes data of the 8 subjects for one representative experimental session (third session). Assistive torque, normalized as explained in section (3.4.3), is also reported. Phase portraits were represented plotting values of the mean velocity ${\bar{\dot{q}}}_{m}$ vs. the corresponding value of the mean angle ${\bar{q}}_{m}$ for a full flexion-extension cycle.

The first two rows of Figure 5, as well as the phase portrait of the last row, seem to indicate no significant variations in the kinematic pattern between AR and ZT mode, as quantitatively discussed in detail in section (4.1). The soft limits^1^ imposed to the subjects through the GUI (dashed red lines in the angle graphs) were properly met during the task execution.

The muscle activation patterns appeared to be maintained between the two modalities with variations on the amplitude due to the action of the controller. Differences in EMG activity will be presented in section (4.2).

### 4.1. Controller Effect on Task Kinematics

To analyze the differences between mean curves of knee joint angle and angular velocity in AR and ZT mode, the cross-correlation coefficients ρ were calculated as described in section (3.4.5). A very high correlation between curves in AR and ZT mode was found for both angle and velocity patterns, as demonstrated by the value of ρ greater than 0.99 in 100% of the tests. Statistical analysis described in section (3.4.6) was adopted on the distributions of the angular velocity over the full cycle ${\dot{q}}_{k,m-fc}^{RMS}$ and on the distributions of velocity in extension phase ${\dot{q}}_{k,m-ex}^{RMS}$ and in flexion phase ${\dot{q}}_{k,m-fl}^{RMS}$ (with *k* = 1, …, *N*_*m*_).

**Figure 6:**
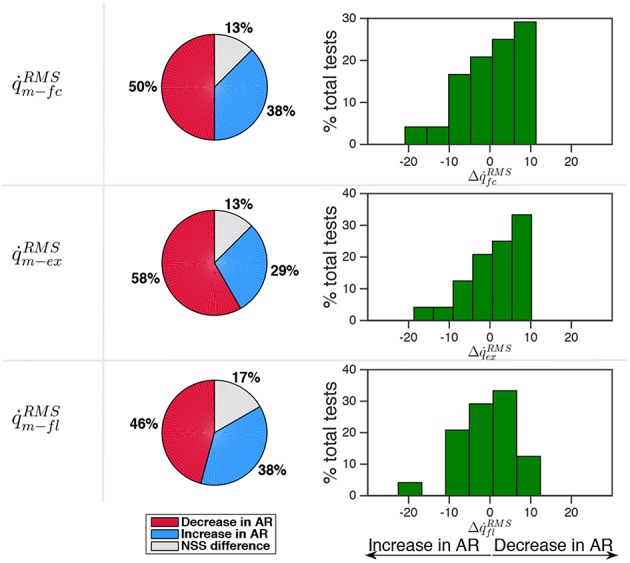
Comparison between velocity in AR and ZT mode expressed as percentage of occurrence of increase/decrease and of NSS difference. Data is related to the three measured sessions.

Results reported in Figure 6 demonstrate that subjects were prone to decrease the velocity of movement in AR mode. In 50% of the tests ${\bar{\dot{q}}}_{ZT-fc}^{RMS}>{\bar{\dot{q}}}_{AR-fc}^{RMS}$ was found while in 38% of the tests the opposite condition occurred. Velocity distributions did not differ in a statistically significant way in 13% of the tests. Even though there is a tendency to reduce the motion velocity in AR mode, the percentage variation $\Delta {\bar{\dot{q}}}_{fc}^{RMS}$ felt in a narrow range of ±10% in 92% of the performed tests. This effect is more evident if the velocity curve is decomposed by considering extension phase ($\dot{q}<0$) and flexion phase ($\dot{q}>0$).

During extension the decrease of velocity in AR mode occurred in 58% of the tests while the increase occurred in 29% of the tests. During flexion no evident trend was identified. It is worth noticing that during the oscillation cycle a single energy parcel provided an amount of energy with respect to the total energy so that ϵ = 19% on average, thus demonstrating that the controller did account for the total energy cost of the task with a non negligible contribution.

### 4.2. Controller Effect on Muscular Activity

**Figure 7:**
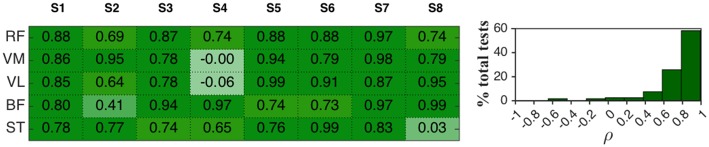
(**Left**) Cross-correlation coefficients ρ between mean EMG activity in AR mode and ZT mode of 8 healthy subjects (session 3). (**Right**) Frequency distribution of ρ expressed in percentage of the total analyzed tests.

Cross-correlation coefficients ρ were also calculated for EMG curves. In Figure 7 the results for the third session were representatively reported, while the histograms on the right represent the frequency distribution for all the tests performed in the three sessions. In 58.3% of the tests ρ values felt in the range 0.79–0.99 while in 25.8% it was in the range 0.58–0.79. Hence, in about 84% of the tests the mean EMG patterns in ZT and AR mode can be considered highly correlated, meaning that average EMG patterns during the flexion-extension cycle tend to remain unaltered during AR mode with respect to ZT.

Values of ρ < 0.58 were found in 10% of the tests for extensor muscles and in 23% of the tests for flexor muscles.

**Figure 8:**
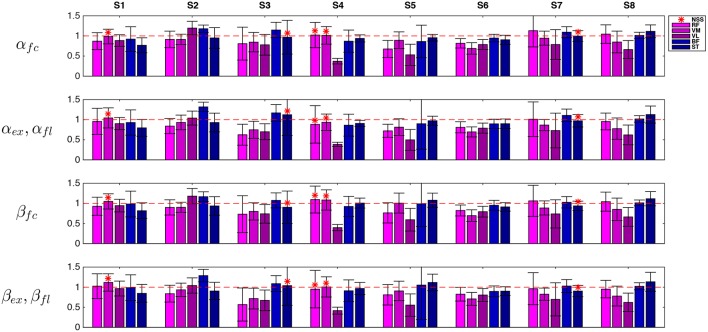
Representative data (session 3) of RMS muscular activity in AR mode normalized with respect to unassisted ZT mode. Flexion quantities α_*fl*_ and β_*fl*_ are considered for flexor muscles while extension quantities α_*ex*_ and β_*ex*_ are considered for extension muscles. Extensor muscles (RF, VM, and VL) are indicated with violet tones and flexor muscles (BF, ST) are indicated with blue tones. NSS data is marked by asterisks.

The ratio between EMG activity in AR and EMG activity in ZT, α_ϕ_ in (8) and the same ratio for the EMG activity normalized by joint velocity, β_ϕ_ in (9), are reported in Figure 8 (NSS data are indicated with red asterisks). Values of α_ϕ_ were below 1 for most of the cases, meaning that the controller effectively caused a decrease of EMG activity.

Nevertheless, to demonstrate that a reduction in muscular activity was not due to a performance decay, and in particular to a decrease in the task velocity, EMG data was normalized with respect to the angular velocity as explained in (9). Normalization was calculated on the full cycle and on extension and flexion phases separately. It is worth noting that extension was performed mainly against gravity while flexion was mainly helped by gravity.

**Figure 9:**
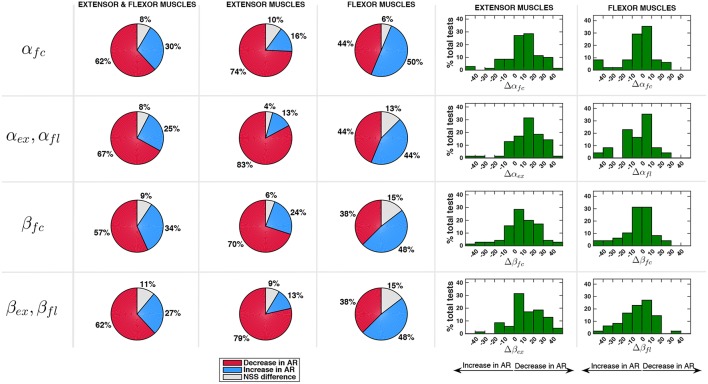
Comparison between EMG activity in AR and ZT mode expressed as percentage of occurrence of increase/decrease and of NSS difference. Percentage variation of muscular activity in AR mode on extensor and flexor muscles was calculated as Δα_ϕ_ = (1 − α_ϕ_)100 and Δβ_ϕ_ = (1 − β_ϕ_)100. In the second and third column of the pie charts flexion quantities α_*fl*_ and β_*fl*_ are considered for flexor muscles while extension quantities α_*ex*_ and β_*ex*_ are considered for extension muscles.

The modifications in the velocity-normalized EMG activity experienced in AR mode with respect to the ZT mode, and possible non-statistically significant differences, are reported in Figure 9 considering all the muscles and then splitting flexor and extensor muscles. Percentage variations of α_ϕ_ and β_ϕ_ were calculated as Δα_ϕ_ = (1 − α_ϕ_)100 and Δβ_ϕ_ = (1 − β_ϕ_)100 and were reported in histograms indicating the frequency distribution with a variation range of 10%. In the second and third column of the pie charts extension quantities α_*ex*_ and β_*ex*_ are considered for extensor muscles while flexion quantities α_*fl*_ and β_*fl*_ are considered for flexor muscles.

Considering the activity over the full cycle α_*fc*_ of all the muscles, the decrease of EMG activity was more frequent (about 60%) than the increase (about 30%). Moreover, a reduction of the EMG activity of the extensor muscles in a number of cases greater than 70% was experienced. For the EMG activity of flexor muscles, on the contrary, a clear decrease was not evident. Indeed, data show that Δα_*fc*_ on extensor muscles was mostly comprised in the range 0–20% while Δα_*fc*_ on flexor muscles was equally distributed in the range -10 to 10%. Analyzing the activity of extensor muscles only during extension phase (α_*ex*_) and flexor muscles only during flexion phase (α_*fl*_), the reduction of muscular activity on extensor muscles was even more evident (frequency of 83%, with a reduction greater than 20% in about 33% of the cases). However, when an increase of EMG was detected (13% of the cases), it was very limited and mostly comprised in the range -10 to 0%. Even focusing only on flexion phase, flexor muscles activity did not appear to reduce, cases of increase of muscular activity in AR and cases of decrease in AR resulted to be equally distributed (44%).

Considering the normalization of the muscular activity with respect to velocity (β_ϕ_), Figure 9 shows that extensor muscles decreased their activity in 70% of the cases within the full cycle and in 79% of the cases during extension. Flexor muscles showed an increase of EMG activity in 48% of the cases and a decrease in 38% of the cases.

**Figure 10:**
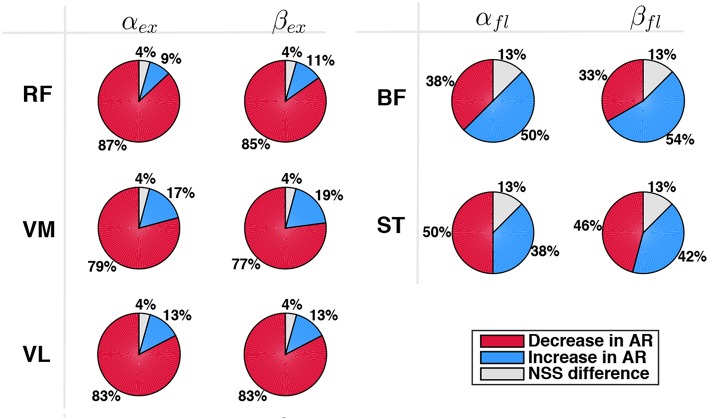
Comparison between EMG activity in AR and ZT mode expressed as percentage of occurrence of increase/decrease and of NSS difference considered for single extensor muscles (RF, VM, VL) during extension (α_*ex*_,β_*ex*_) and for flexor muscles (BF, ST) during flexion (α_*fl*_,β_*fl*_).

Finally, each muscle was analyzed separately, with particular attention to extensor muscles during extension phase and flexor muscles during flexion phase. Pie charts in Figure 10 represent the percentage of cases of decrease/increase and of non-statistically significant differences. Considering α_ϕ_, *RF* turned out to be the muscle which most benefits from the robotic aid since a decrease of its activity in AR mode was experienced in 87% of the cases; *VL* and *VM* activity decreased in 83% and 79% of the cases, respectively. Considering flexor muscles, *ST* decreased its activity in 50% of the cases and increased it in 38% of the cases. Therefore, *BF* was the only muscle that did not benefit from the robotic assistance. Similar results were obtained for normalized data β_ϕ_ (very slight differences were found with respect to non-normalized data α_ϕ_).

## 5. Discussion

The aim of this work was to provide a proof-of-concept of the effectiveness of a novel control scheme for torque-controlled exoskeletons assisting cyclic tasks. The proposed controller is based on the concept of switching intervention, able to provide the minimum amount of energy required to feed a limit cycle thus minimally interfering with user's natural motion. Using a set-up including a 1-DOF exoskeleton to assist oscillations of the leg around the knee axis of rotation in the sagittal plane, the SAC was validated through experiments on 8 healthy subjects performing flexion/extension motions in unassisted and assisted conditions. Two key features of the SAC were assessed, namely the capability of: (i) minimizing the perturbations to the user's original unassisted kinematic status; (ii) providing effective physical support thus reducing the user's muscular effort in performing the task.

### 5.1. Controller Effect on Task Kinematics

We verified that motion kinematic features were not altered based on the following results:

- Cross-correlation coefficients calculated for both knee joint angle and angular velocity curves in AR and ZT modes were greater than 0.99 in 100% of the tests (Figure 7), thus demonstrating the preservation of the user's natural motion;
- No significant alterations, as a consequence, were found on phase portraits of AR and ZT mode (Figure 5);
- Despite a reduction of the velocity profile amplitude was experienced in most of the cases, RMS value over the flexion-extension cycle felt in a narrow range of ±10% in 92% of the tests performed (Figure 6).

This result, as explained in section (2.1), was achieved by minimizing the changes to the motion velocity through the proper timing of robotic intervention, i.e., by a minimal assistance concentrated in the time interval when angular momentum is maximum.

### 5.2. Controller Effect on Muscular Activity

We verified that physical assistance was delivered effectively by assessing the reduction of user's muscular effort based on the following results in AR mode with respect to baseline ZT mode:

- Muscle activation patterns were not significantly altered since the cross-correlation coefficient between AR and ZT EMG curves was greater than 0.58 in 84% of the tests. Poor correlation between AR and ZT mode was found in 10% of the tests on extensor muscles and in 23% of the tests on flexor muscles. This was likely caused by low EMG quality of flexor muscles due to mechanical/electrical artifacts (cables and electrodes motion) and to reduced skin impedance (skin sweating).
- Data on effort decrease of extensor muscles clearly highlighted the effectiveness of the proposed controller (extension was the most demanding phase since mainly performed against gravity), despite a slight increase of flexors activity. Even in the case of increase in extensors EMG, the percentage of increase was found to be very limited (Figure 9). Considering muscular activity of extensor muscles during the extension phase α_*ex*_, in 83% of the tests there was a decrease of EMG activity and the frequency distribution had a peak on the range of variation 10–20%; only in 13% of the tests there was an EMG increase; moreover, in 75% of these cases the increase was basically negligible (0–10%). Considering muscular activity of flexor muscles during the flexion phase, there were no apparent trends (percentages were almost equivalent for both conditions). The most likely causes were the noisy signal due to mechanical artifacts (i.e., subjects sitting on electrodes) and the asymmetrical range of oscillation [−31°, 19°], which made the task most challenging in extension with respect to flexion.
- The reduction of EMG activity was not amenable to a reduction in the velocity of the task. This aspect was confirmed by the analysis of data normalized with respect to the velocity (Figure 9), that showed again a reduction of EMG in AR with respect to the ZT mode.

### 5.3. Controller Application

The use of the SAC is expected to be potentially extended to other human joints and to different motor tasks. The specific features of the controller particularly fit with applications to exoskeleton-assisted walking. The controller could easily allow the user to keep the mastery over the robot, which in turn would provide assistance only by seconding the subject's intended motion and by adapting automatically to his walking style and preferred speed. Moreover, safety could be intrinsically guaranteed due to the limited mechanical energy transferred to the subject and to the absence of any potentially constraining predefined trajectory. Brief switching energy parcels delivered in the most convenient phases of gait could be the most appropriate way of providing assistance without destroying the natural efficient pendular nature of legs and body motion during walking. Indeed, studies on passive walkers (Collins et al., 2005) demonstrated that locomotion significantly relies on intrinsic dynamics. Hence, the need to minimally perturb this structural optimization mechanism makes the SAC, with its minimal assistive intervention and with its specific applicability to periodic motions, particularly suitable for walking assistance.

## 6. Conclusions

The proposed controller provides assistive inputs that intermittently inject energy into the human-robot compound system to maintain a stable limit cycle, i.e., to feed oscillation dynamics with the minimum required amount of energy, and to minimize perturbations thanks to a specific delivery timing. The energy injection is pursued in specific phases when the alteration to the system status can be minimized (i.e., when the angular momentum is maximum). The controller was tested on a group of healthy subjects performing knee flexion-extension assisted by a compliant exoskeleton. EMG activity of the major muscles supporting knee flexion-extension was assessed when the subjects were oscillating the leg with a soft constraint displayed on a screen. In most of the tests the controller was effective in minimizing changes to the kinematic status of the system and in reducing the muscular activity while the subject was assisted. Future work will be devoted to adapt the level of the torque to the anthropometric characteristics of the subjects to magnify the assistive effect, and also to assess possible reductions of metabolic cost. Moreover, the use of the controller will be extended to other human joints, with proper modifications to the algorithm to take into account possible issues arising from more complex dynamics during human-robot interaction and from potential saturation effects. Future activities will be also dedicated to a more extensive characterization of the controller parameters with the aim to optimize the delivered energy and to take into account possible effects due to the changes of the torque waveform and/or to the timing of the assistance.

## Ethics Statement

This study was carried out in accordance with the recommendations of the Ethics Committee of Università Campus Bio-Medico di Roma with written informed consent from all subjects. All subjects gave written informed consent in accordance with the Declaration of Helsinki. The protocol was approved by the Ethics Committee of Università Campus Bio-Medico.

## Author Contributions

NT and AS implemented the controller based on the concept developed by AS. NT, DA, and DF designed the study and the specific experiments. SV and CD performed the experiments. SV, CD, and IP performed the data analysis with contributions from NT and DF. NT and SV wrote the manuscript. DA and DF participated in the design and drafting of the manuscript. NT, DA, and DF were involved in results interpretation and critical revision of the study. All authors read and approved the final manuscript.

### Conflict of Interest Statement

The authors declare that the research was conducted in the absence of any commercial or financial relationships that could be construed as a potential conflict of interest.

## Footnotes

^1^Subjects were asked to consider the limits only as a graphical reference. Limits could be exceeded so no real constraints were actually imposed.
